# Association of Periodontitis and Various Genotypes of Human Papillomavirus in Oral Rinse Specimens

**DOI:** 10.7759/cureus.60190

**Published:** 2024-05-13

**Authors:** Hamad Aldhubaiei, Majdah M Alzuabi, Yousef Marafi, Fatemah Alzalzalah, Mohammad Aljalahmah, Zuhair S Natto

**Affiliations:** 1 Dentistry, Ministry of Health, Kuwait, KWT; 2 Ear, Nose and Throat, Ministry of Health, Kuwait, KWT; 3 Dental Public Health, King Abdulaziz University, Jeddah, SAU

**Keywords:** genotype, hpv, human papilloma virus, periodontitis, infection

## Abstract

Objectives

To evaluate the relationship between the presence of human papillomavirus (HPV) genotypes in oral rinse samples and periodontitis.

Materials and methods

This cross-sectional study utilized data from the National Health and Nutrition Examination Survey (NHANES) conducted in 2013-2014. The primary outcome was the periodontal status, categorized as either no periodontitis or mild periodontitis (combined) versus moderate to severe periodontitis (combined). The primary variable of interest was the presence of different HPV types in oral rinse specimens. Several confounders were selected based on previous evidence that demonstrated a potential association between HPV infectivity and periodontal disease.

Results

The final sample included 3103 participants. HPV genotypes 6, 35, 39, 55, 59, 71, 72, and 73 showed a statistically significant association with at least one of the periodontal statuses (p-value < 0.05). The presence of any HPV genotype was highly significantly associated with periodontal status, with a p-value of <0.001. Multivariable logistic regression analysis revealed statistically significant associations of HPV 6 and HPV 16 with periodontitis, adjusted for age, gender, diabetes, smoking, race, federal poverty level, last dental visit, and education level.

Conclusion

Our study highlights a potential link between periodontitis and specific HPV genotypes, such as HPV 6 and HPV 16, in oral rinse specimens. This suggests a complex interplay between periodontal disease and oral HPV infections, underscoring the need for further research to address public health concerns and inform preventive and treatment strategies.

Clinical relevance

Identifying a link between periodontitis and specific HPV genotypes, such as HPV 6 and HPV 16, in oral rinse samples could prompt early screening and tailored treatment approaches. This underscores the importance of oral health promotion and targeted interventions to address both conditions and improve overall patient outcomes.

## Introduction

Periodontitis, a multifaceted chronic oral disease, involves the interplay among hard tissues such as cementum and bone, soft tissues such as epithelial cells and connective tissue, and a diverse biofilm comprised of bacteria, fungi, and viruses, alongside the immune system's reaction to this biofilm [1-3]. This condition is associated with several chronic inflammatory ailments like diabetes and cardiovascular disease [4]. In periodontitis, there is an ongoing discharge of inflammatory cytokines and biomarkers, which detrimentally impact overall health and are linked to alterations in epithelial barrier function [4].

There is a hypothesis suggesting that the periodontal pocket could enhance the colonization and persistence of various bacteria and viruses, including SARS-CoV-2 [5]. Among these, human papillomavirus (HPV) in the oral mucosa is highlighted as potentially serving as a reservoir for latent HPV [1, 3, 6]. However, the precise infection mechanism remains unclear, and whether the presence of biofilm and chronic inflammation in periodontitis, leading to reduced epithelial barrier protection, correlates with oral HPV infectivity remains to be elucidated. This study aims to identify the HPV genotypes present in oral rinse specimens (ORS) associated with periodontitis. The null hypothesis suggests that the odds for any HPV genotype in ORS are equivalent between individuals with and without periodontitis. Previous studies on this subject have produced conflicting results, with some limited by reliance on self-reports for periodontitis identification or using self-reported oral health as the periodontal parameter. Additionally, limited sample sizes or select patients in a hospital setting have been observed. This study adds to the existing literature by presenting findings from a large, nationally representative sample. Clinical assessment of all teeth was conducted, and the definition of periodontitis aligns with the criteria for population surveillance established by the CDC, developed in collaboration with the American Academy of Periodontology (AAP) [7].

## Materials and methods

Study design and data source

A cross-sectional study design was utilized to explore the relationship between the positivity of different types of HPV in ORS and periodontitis. The data were extracted from the National Health and Nutrition Examination Survey (NHANES), which surveys the non-institutionalized civilian population using a stratified, multistage probability sampling methodology. This analysis employed secondary data from an open dataset; hence, ethical approval was not required. However, the original NHANES study received approval from the National Center for Health Statistics Institutional Review Board (Protocols #98-12, #2005-06, and #2011-17). Before participation, individuals provided written informed consent. Trained interviewers conducted household surveys and facilitated participants' physical examinations at NHANES mobile examination centers. Eligible NHANES participants ranged in age from 14 to 69 years. More details about NHANES can be accessed at http://www.cdc.gov/nchs/nhanes.htm.

Study sample

The study sample comprised a subset of the NHANES dataset from the years 2013-2014. To be included, participants needed to have both comprehensive periodontal and HPV data. The dataset focused on participants aged 30-69 years, excluding edentulous individuals and those under the age of 30.

Outcome/Dependent variable

The study’s primary outcome was categorized periodontal status: no periodontitis or mild (combined) versus moderate or severe periodontitis (combined). The definition of periodontitis adhered to the criteria for population surveillance established by the CDC and AAP [7]. Mild periodontitis was characterized by either one periodontal site with a probing depth (PD) of ≥5 mm or at least two interproximal sites with ≥3 mm clinical attachment loss (CAL) and at least two interproximal sites with a PD of ≥4 mm, not on the same tooth. Moderate periodontitis entailed at least two interproximal sites with PD ≥5 mm not on the same tooth or at least two interproximal sites with CAL of ≥4 mm, not on the same tooth. Severe periodontitis was characterized by at least one interproximal site with a PD of ≥5 mm and at least two interproximal sites on different teeth with CAL of ≥4 mm. Due to sample size considerations, the data were dichotomized into no periodontitis and mild versus moderate and severe periodontitis. Periodontal assessments were conducted by licensed dentists using halogen illumination, mirrors, and Hu-Friedy™ periodontal probes. Further details are accessible on the NHANES website.

Main exposure

The primary variable of interest was the positivity of different HPV genotypes in ORS. Participants provided HPV samples by swishing and gargling 10 mL of mouth rinse or saline for 30 seconds during oral evaluations. These samples were stored at 4°C and analyzed for HPV using polymerase chain reaction. Additional information is available on the NHANES website.

Other variables/potential confounders

Sociodemographic variables were examined as potential confounders, including gender (male vs. female), race/ethnicity (Non-Hispanic white, Non-Hispanic black, Mexican American, or Other), education (0 to 11th grade, high school graduates, above high school), Federal poverty level (<100%, 100% to 199%, 200% to 399%, ≥400%), smoking status (current or former smoker vs. never smoker), diabetes status (diabetic or borderline vs. none), and time since last dental visit (≤6 months, >6 months to one year, more than one year to 2 years, >2 years or never). These variables were chosen based on previous research indicating their correlation with both HPV infectivity and periodontal disease.

Sample size and statistical analysis

This is a secondary data analysis, and the entire dataset meeting the inclusion criteria was included. The final sample consisted of 3,103 participants. NHANES employs a sophisticated study design, incorporating sample weights in the statistical analyses conducted for this study. These analyses included descriptive statistics (frequency and percentage) as well as unadjusted and adjusted logistic regression models focused on HPV. The fully adjusted logistic regression model accounted for age, gender, diabetes, smoking, race, federal poverty level, last dental visit, and education level, alongside the primary variable of interest, HPV genotype. Data analysis was conducted using SAS 9.3® software (Cary, NC, USA).

## Results

Demographics of study sample

Table 1 displays the demographic characteristics of the study sample based on the severity of periodontitis. The variables include several potential confounders such as age, gender, diabetes status, smoking habits, race, federal poverty level, last dental visit, and education level. Each variable is further categorized based on the severity of periodontitis: Healthy/Mild, Moderate, and Severe. Individuals aged 50-64, males, non-Hispanic blacks, and those with lower education levels (0 to 11th grade) show a higher prevalence of severe periodontitis compared to other groups (p-value < 0.001).

**Table 1:** Demographic characteristics of study sample. *P-value < 0.05.

Variable	Healthy/Mild periodontitis N=1842 n(%)	Moderate periodontitis N=985 n(%)	Severe periodontitis N=276 n(%)	P-value
Age				<0.001*
30-34	331(18.0)	109(11.1)	13 (4.7)	
35-49	832 (45.2)	349 (35.4)	71 (25.7)	
50-64	561 (30.5)	396 (40.2)	159 (57.6)	
>64	118 (6.4)	131 (13.3)	33 (12.0)	
Gender				<0.001*
Males	768 (41.7)	546 (55.4)	186 (67.4)	
Females	1074 (58.3)	439 (44.6)	90 (32.6)	
Diabetes				0.419
Diabetic or borderline	177 (9.6)	107 (10.9)	32 (11.6)	
Non diabetic	1662 (90.4)	877 (89.1)	244 (88.4)	
Smoking				0.445
Current or former	655 (41.6)	365 (44.3)	103 (43.1)	
Never	919 (58.4)	459 (55.7)	136 (56.9)	
Race				<0.001*
Mexican American	381 (20.7)	288 (29.2)	78 (28.3)	
Non-Hispanic white	845 (45.9)	309 (31.4)	75 (27.2)	
Non-Hispanic black	303 (16.4)	239 (24.3)	93 (33.7)	
Others	313 (17.0)	149 (15.1)	30 (10.9)	
Federal poverty level				<0.001*
<100%	232 (13.6)	254 (28.2)	90 (35.7)	
100% to 199%	343 (20.1)	254 (28.2)	70 (27.8)	
200% to 399%	472 (27.6)	228 (25.3)	62 (24.6)	
≥400%	662 (38.7)	165 (18.3)	30 (11.9)	
Last dental visit				0.359
≤6 months	879 (47.8)	463 (47.1)	139 (50.4)	
>6 months -1 year ago	247 (13.4)	160 (16.3)	38 (13.8)	
>1 year - 2 years ago	177 (9.6)	100 (10.2)	29 (10.5)	
>2 years, or never	534 (29.1)	261 (26.5)	70 (25.4)	
Education level				<0.001*
0 to 11th grade	229 (12.4)	277 (28.2)	106 (38.4)	
High school graduate	319 (17.3)	270 (27.4)	80 (29.0)	
Above high school	1294 (70.2)	437 (44.4)	90 (32.6)	

Association between HPV genotypes and periodontitis

The distribution of HPV genotypes across different periodontal statuses is presented in Table 2. HPV genotypes 6, 35, 39, 55, 59, 71, 72, and 73 have a statistically significant association with at least one of the periodontal statuses (p-value < 0.05). The HPV genotype "any" shows a highly significant association with periodontal status, with a P-value of < 0.001.

**Table 2:** Association between HPV genotypes and several periodontal statuses. *p-value < 0.05. NA: Not applicable; HPV: Human papillomavirus.

Variable	Healthy/Mild periodontitis N=1842 n (%)	Moderate periodontitis N=985 n (%)	Severe periodontitis N=276 n (%)	P-value
HPV 06	3 (0.2)	7 (0.7)	3 (1.1)	0.011*
HPV 11	0 (0)	1 (0.1)	0 (0)	0.406
HPV 16	18 (1.0)	7 (0.7)	2 (0.7)	0.74
HPV 18	3 (0.2)	2 (0.2)	1 (0.4)	0.538
HPV 26	0 (0)	0 (0)	0 (0)	NA
HPV 31	1 (0.1)	2 (0.2)	0 (0.0)	0.414
HPV 33	3 (0.2)	3 (0.3)	1 (0.4)	0.403
HPV 35	6 (0.3)	3 (0.3)	4 (1.4)	0.040*
HPV 39	6 (0.3)	0 (0)	3 (1.1)	0.012*
HPV 40	0 (0)	1 (0.1)	0 (0)	0.406
HPV 42	1 (0.1)	1 (0.1)	0 (0)	1
HPV 45	0 (0)	1 (0.1)	0 (0)	0.406
HPV 51	4 (0.2)	1 (0.1)	0 (0)	0.789
HPV 52	2 (0.1)	2 (0.2)	1 (0.4)	0.361
HPV 53	7 (0.4)	7 (0.4)	1 (0.4)	0.439
HPV 54	2 (0.1)	0 (0)	0 (0)	0.623
HPV 55	5 (0.3)	8 (0.8)	5 (1.8)	0.004*
HPV 56	10 (0.5)	2 (0.2)	1 (0.4)	0.381
HPV 58	3 (0.2)	2 (0.2)	0 (0)	0.758
HPV 59	8 (0.4)	11 (1.1)	0 (0)	0.034*
HPV 61	8 (0.4)	12 (1.2)	2 (0.7)	0.061
HPV 62	13 (0.7)	14 (1.4)	5 (1.8)	0.081
HPV 64	0 (0)	0 (0)	0 (0)	NA
HPV 66	13 (0.7)	4 (0.4)	1 (0.4)	0.536
HPV 67	1 (0.1)	0 (0)	0 (0)	0.71
HPV 68	1 (0.1)	1 (0.1)	2 (0.7)	0.063
HPV 69	0 (0)	3 (0.3)	0 (0)	0.081
HPV 70	2 (0.1)	0 (0)	0 (0)	0.623
HPV 71	1 (0.1)	4 (0.4)	2 (0.7)	0.018*
HPV 72	4 (0.2)	9 (0.9)	4 (1.4)	0.006*
HPV 73	1 (0.1)	0 (0)	2 (0.7)	0.022*
HPV 81	6 (0.3)	3 (0.3)	0 (0)	0.64
HPV 82	1 (0.1)	0 (0)	0 (0)	0.71
HPV 83	5 (0.3)	4 (0.4)	1 (0.4)	0.702
HPV 84	11 (0.6)	5 (0.5)	2 (0.7)	0.905
HPVCP6108	3 (0.2)	7 (0.7)	1 (0.4)	0.064
HPV IS39	2 (0.1)	1 (0.1)	0 (0)	0.862
HPV any	117 (6.4)	94 (9.5)	35 (12.7)	<0.001

Table 3 presents the results of univariate and multivariable logistic regression analyses between different HPV genotypes and the presence of moderate/severe periodontitis. HPV 6, HPV 55, HPV 16, HPV 61, and HPV 71 show statistically significant associations with moderate/severe periodontitis (p-value <0.05) in the univariate analysis. In the multivariable analysis, HPV 6 and HPV 16 maintain significant associations with periodontitis after adjustments for age, gender, diabetes, smoking, race, federal poverty level, last dental visit, and education level.

**Table 3:** Univariate and multivariable logistic regression between HPV genotypes and several periodontal status *p-value <0.05. OR: Odds ratio; CI: Confidence interval; NA: Not applicable; HPV: Human papillomavirus. ** Adjusted for age, gender, diabetes, smoking, race, federal poverty level, last dental visit and education level.

Variable	Univariate model Moderate/Severe periodontitis N=1261 OR (95% CI)	P-value	Multivariable model** Moderate/Severe periodontitis N=1261 OR (95% CI)	P-value
HPV 06	0.016 (0.056-0.743)	0.016*	0.253 (0.064-0.995)	0.049*
HPV 11	NA		NA	
HPV 16	1.373 (0.615-3.066)	0.44	3.382 (1.143-10.003)	0.028*
HPV 18	0.684 (0.138-3.395)	0.642	1.172 (0.164-8.404)	0.874
HPV 26	NA		NA	
HPV 31	0.342 (0.031-3.775)	0.381	NA	
HPV 33	0.513 (0.115-2.294)	0.382	0.466 (0.071-3.069)	0.427
HPV 35	0.585 (0.196-1.746)	0.337	0.816 (0.200-3.328)	0.777
HPV 39	1.370 (0.342-5.490)	0.656	3.702 (0.380-36.040)	0.26
HPV 40	NA		NA	
HPV 42	0.684 (0.043-10.952)	0.789	1.884 (0.091-38.858)	0.682
HPV 45	NA		NA	
HPV 51	2.742 (0.306-24.562)	0.367	NA	
HPV 52	0.456 (0.076-2.732)	0.39	1.339 (0.172-10.436)	0.781
HPV 53	0.597 (0.216-1.652)	0.321	1.041 (0.335-3.231)	0.945
HPV 54	NA		NA	
HPV 55	0.261 (0.093-0.735)	0.011*	0.662 (0.184-2.386)	0.529
HPV 56	2.289 (0.629-8.33)	0.209	3.408 (0.618-18.810)	0.159
HPV 58	1.027 (0.171-6.155)	0.977	0.374 (0.048-2.908)	0.347
HPV 59	0.496 (0.199-1.236)	0.132	1.418 (0.398-5.054)	0.59
HPV 61	0.389 (0.163-0.929)	0.034*	0.667 (0.237-1.874)	0.442
HPV 62	0.465 (0.229-0.944)	0.034*	1.401 (0.519-3.777)	0.506
HPV 64	NA		NA	
HPV 66	1.785 (0.635-5.021)	0.272	1.140 (0.279-4.655)	0.856
HPV 67	NA		NA	
HPV 68	0.228 (0.024-2.192)	0.2	0.685 (0.048-9.875)	0.781
HPV 69	NA		NA	
HPV 70	NA		NA	
HPV 71	0.114 (0.014-0.945)	0.044*	NA	
HPV 72	0.209 (0.068-0.642)	0.006*	0.514 (0.137-1.931)	0.324
HPV 73	0.342 (0.031-3.775)	0.381	0.769 (0.056-10.556)	0.844
HPV 81	1.370 (0.342-5.490)	0.656	2.185 (0.400-11.938)	0.367
HPV 82	NA		NA	
HPV 83	0.684 (0.198-2.367)	0.548	3.316 (0.608-18.011)	0.166
HPV 84	1.076 (0.416-2.784)	0.88	1.068 (0.342-3.333)	0.909
HPV CP6108	0.256 (0.068-0.965)	0.044*	0.399 (0.063-2.518)	0.328
HPV IS39	1.370 (0.124-15.120)	0.797	1.093 (0.054-22.066)	0.954
HPV any	0.595 (0.458-0.773)	<0.001*	1.016 (0.729-1.416)	0.927

This table provides valuable insights into the potential relationship between specific HPV genotypes and different levels of periodontal disease, highlighting the need for further research to understand the mechanisms underlying this association.

## Discussion

This study examines the association of specific HPV genotypes in ORS with periodontitis, adjusting for several factors such as age, gender, diabetes, smoking, race, federal poverty level, last dental visit, and education level. The findings suggest a potential link between the presence of HPV in ORS and periodontitis, contributing valuable insights to existing knowledge regarding the role of periodontitis in HPV genotypes found in ORS. Unlike previous NHANES studies that did not find significant associations and assessed HPV broadly [8], this cross-sectional study highlights a connection between periodontitis and oral HPV.

A recent review of 12 articles investigating the potential link between periodontitis and HPV presented conflicting and inconclusive findings [6]. Nevertheless, several studies did suggest a connection between oral HPV infection and periodontitis [6,9-11]. Discrepancies among these studies primarily arise from differences in methodology and definitions used to assess periodontitis and oral HPV infection status [6]. Notably, bleeding on probing (BOP), a classic indicator of periodontal inflammation, emerged as a significant factor in routine periodontal assessments and was closely correlated with active periodontal disease [6]. Additionally, HPV16 was found to be more frequently associated with periodontitis in elderly women [10].

Various studies have reported positive associations between periodontitis and HPV. For instance, Hormia M et al. discovered high-risk HPV in 26% of gingival biopsy specimens from individuals with periodontitis, suggesting the periodontal pocket as a potential reservoir for HPV [3]. Other researchers using different methods also found HPV in gingival tissue, indicating the gingival epithelium as another possible HPV reservoir [12]. Tezal M et al. linked HPV-positive status with alveolar bone loss in tongue cancer biopsy specimens, indicating an association between periodontal status and HPV infection [13-14].

HPV can be divided into high-risk types such as HPV 16, 18, 31, 33, 35, 39, 45, 51, 52, 56, 58, and 59, and low-risk types which may cause warts on or around the genitals, anus, mouth, or throat [15]. In our study, we found some high-risk HPV genotypes such as HPV16, HPV 35, HPV 39, and HPV 59, which might be related to periodontal disease. Moreover, HPV16 is responsible for most HPV-related cancers [15].

The most common HPV genotype that can potentially affect periodontal disease was HPV16 (15.0% of cases with periodontal disease), followed by HPV11 (10.0%) and HPV6 (25.0%), suggesting that the periodontal pocket may act as a reservoir for HPV infection [16]. Another study reported a prevalence of HPV16 among the study sample to be 67.0% for periodontitis [17]. In our study, HPV16 was the most prevalent among the study population and specifically among the healthy/mild periodontitis group, not with the advanced cases. The possible justification is that HPV16 was the most common high-risk HPV genotype among a similar population of 3,196 participants in a US healthy adult population (12.4% among men and 8.6% among women), so it is part of the healthy/mild periodontitis flora [6]. However, studies could not find a link between HPV16 and periodontitis [1,17]. Moreover, one study found no HPV16 in cases with periodontitis or controls, which concluded there was no association between HPV16 and periodontal disease [1].

Certain strains of HPV, particularly HPV16, have been detected in oral tissues, periodontal pockets, and lesions, suggesting a potential association with periodontal inflammation and tissue destruction [1,3,6]. Moreover, the inflammatory response triggered by HPV infection may exacerbate the progression of periodontal disease by disrupting the delicate balance of oral microbial communities and immune responses [1,3,6]. Conversely, periodontal disease, characterized by chronic inflammation and tissue damage in the gums and supporting structures of the teeth, may create a favorable environment for viral persistence and replication [1,3,6]. Periodontal pathogens can lead to periodontal pocket formation through the inflammatory response, which will act as a source of inflammatory cytokines, bacteria, and viruses in saliva, creating an ideal environment for HPV [17], which also can promote inflammation that could increase the severity of periodontitis [18]. HPV also can gain access to the body by attacking the basal cells of the epithelium in the periodontal pocket [19]. Thus, the association between periodontal disease and HPV could be direct through the oral microbiota or indirect through the stimulation of chronic inflammation [20]. At the same time, this relationship seems to be bidirectional, with HPV potentially contributing to periodontal disease through inflammatory secretion, or periodontal disease acting as a reservoir for HPV [21]. Additionally, both HPV infection and periodontal disease have been independently linked to an increased risk of certain cancers, suggesting a complex interplay between viral infections, oral health, and systemic health outcomes [6,22].

This study has several strengths, such as the use of clinical periodontal data from the NHANES 2013-2014, avoiding potential social biases associated with self-reported data. Moreover, the periodontitis data included comprehensive evaluations of all teeth rather than partial mouth assessments, ensuring a robust analysis based on an established definition. Polymerase chain reaction testing was used to analyze HPV, and the study encompassed a large, nationally representative sample size (N=3103), drawn from a high-quality data source. Despite these strengths, the study's design has limitations in its inability to establish causal or temporal relationships. Additionally, other social determinants or biological factors beyond those considered may confound or modify the observed relationships.

## Conclusions

Periodontitis is a multifaceted condition influenced by various factors. Our study suggests a potential association between periodontitis and the presence of specific HPV6 and HPV16 genotypes in ORSs, as indicated by the adjusted model. This finding underscores the complexity of periodontal disease and its potential interplay with oral HPV infections. Furthermore, the detection of multiple HPV genotypes in oral rinse specimens raises public health concerns and underscores the need to investigate the mechanisms underlying this association and its implications for public health.
